# Editorial: Genomics and Epigenomics of Cancer Immunotherapy: Challenges and Clinical Implications

**DOI:** 10.3389/fonc.2021.704397

**Published:** 2021-07-22

**Authors:** Malak Abedalthagafi

**Affiliations:** King Fahd Medical City, Saudi Genome Project, King Abdulaziz City for Science and Technology, Riyadh, Saudi Arabia

**Keywords:** genomic, epigenomic, cancer, immunotherapy, oncology

Aberrant gene function and altered gene expression are hallmarks of cancer. While the success of cancer immunotherapy has provided a qualitative leap in cancer management, not all patients have not benefited from immunotherapies available today as they fail to achieve complete responses, suffer frequent relapses, or develop life-threatening drug related toxicities. It is now well understood that genetics, epigenetics as well as the immune system plays a vital role in the invasiveness, migration and progression of cancer. Genomic and epigenetic signatures in immune and cancer cells may provide accurate prediction of patients treated with immunotherapeutics. Tumor-associated macrophages are polarized into phenotypes M1 and M2 (1), where the M1 polarized macrophages secrete pro-inflammatory cytokines to eliminate tumor cells, while M2 polarized macrophages secrete anti-inflammatory cytokines and have implications on tumor angiogenesis, metastasis and growth and may result in a worse prognosis (2). Successful cancer immunotherapy will be aided by modulating M2-type macrophage function that predominates human cancers to produce growth-promoting molecules thereby stimulating tumor growth into M1-type, which have the ability to retard cancer growth. This interplay between pro- and anti-inflammatory mechanisms is known to regulate tumor progression and predict cancer susceptibility. The fact that macrophages and thereby, the innate immunity, can be modulated to play a central role in combating cancer is a breakthrough that is imperative for the realization of success in the use of immunotherapy against cancer (3). During cancer progression, neoplastic cells undergo reversible transitions in multiple phenotypic states. Of these, the extremes are defined as the epithelial and mesenchymal phenotypes. Epithelial-to-mesenchymal transition (EMT) remains fundamental to the metastatic process and is associated with changes in the expression of multiple genes, characterized by the downregulation of epithelial markers, and upregulation of mesenchymal markers. Understanding these functional interactions between such EMT-inducing factors and chromatin modulators will provide insights into the mechanisms underlying cancer progression. Furthermore, these studies can enable development of new diagnostic and therapeutic modalities for treating high-grade malignancies (4). Interestingly, EMT is not only controlled at a genetic level, but also at a post-transcriptional level by miRNAs and long noncoding RNAs (lncRNAs). lncRNAs are transcripts >200 nucleotides in length lacking any protein coding potential and are uniquely expressed in differentiated tissues or specific cancer types. Their roles in the modulation of cellular signaling pathways, including those associated with the immune system are only starting to be unraveled. Interestingly, lncRNAs are highly regulated and are restricted more stringently to specific cell types than mRNA (5). lncRNAs are also evolutionarily conserved with regards to the function, secondary structure, and regions of micro homology, despite minimum sequence similarity (6, 7) and have remained oblivious to a rule-based framework for understanding the impact of sequence on function. LncRNAs modulate gene expression, including chromatin modification as well as transcriptional and posttranscriptional processing (8) and have recently gained attention as they can function as a competing endogenous RNA (ceRNA), which can hinder the function of miRNAs in tumors (9), adding another layer of complexity to the mechanisms involved in tumor progression. Several lncRNAs are reported to participate in macrophage polarization while the function of others remains to be unraveled.

Of the 18 manuscripts in this collection, 9 address the role of lcnRNAs in cancers and the interplay between the regulation of lncRNAs, miRNAs, and cellular signaling pathways involving cell migration, proliferation and invasiveness across different cancer subtypes. Wang J et al. explore the role of lncGNAT1-1 in suppression of liver cancer progression by modulating EMT and another manuscript elucidates the role of lncCCAT-1 in promoting EMT, cellular migration and invasion in lung adenocarcinoma by suppressing mir 219-1 (Wang W et al.). Functional and cellular mechanisms involved in migration and invasion of different tumors have been studied for specific lncRNAs in 7 manuscripts (Du et al.; Tu et al.; Ai et al.; Ma et al.; Li et al.; Sun et al.; Jia et al.). The role of lncRNAs derived from macrophage exosomes and their impact on inhibiting the progression of oral squamous cell carcinoma *via* miR-182-5p/FOXO3 pathway has specifically been addressed by Ai et al. and the influence of M2 Polarized Macrophages in Cholangiocarcinoma through modulation of miR-326 and RhoA-ROCK Signaling Pathway has been studied by Tu et al. Immunological infiltration of tumors is the starting point for a successful cancer immunotherapy. 3 manuscripts in this collection address the prognostic value of specific biomarkers by correlating tumor mutational burden and immunological infiltration (Dai et al.; Zhou et al.; Feng et al.). The modulation of tumor microenvironment by BCL9 has been addressed by 2 manuscripts (Wei et al.; Zhu et al.) where the role of BCL 9 in the regulation of the endothelial cell function and fibroblast function has been investigated in the context of tumor microenvironment. Liu et al. explore the role of CD47 expression levels in the progression of endometrial cancer and Zhao et al. explore the role of 22 subsets of tumor-infiltrating immune cells and identify nuclear factors of activated T cells-4 (NFAT4) as the key immune-function related gene associated with poor prognosis in AML mediated *via* recruitment of T regulatory cells (Tregs). Lu et al. explore the role of LINC00675 as a competing endogenous RNA (ceRNA) in HCC cells and its function in restraining metastasis in hepatocellular carcinoma by sponging miR-942-5p. Lastly, the role of TGFβ1 as a prognostic factor in the assessment of tumor immune microenvironment in colon cancer is explored in the study by Wang et al.

The Research Topic of 18 manuscripts covers emerging themes in the field of cancer immunotherapy and we envisage that this collection contributes to the interdisciplinary work involving cellular signaling, miRNAs, genomic and epigenetic characterizations and the implications of lncRNAs across the spectrum of cancer subtypes. These studies on the genomics and epigenetic mechanisms are imperative to the development of novel biomarkers and advance our understanding in elucidating mechanisms involved in immune escape and eventually, help develop improved immunotherapeutic treatment modalities against cancer.

## Author Contributions

The author confirms being the sole contributor of this work and has approved it for publication.

## Conflict of Interest

The author declares that the research was conducted in the absence of any commercial or financial relationships that could be construed as a potential conflict of interest.
